# Immune signature driven by ADT-induced immune microenvironment remodeling in prostate cancer is correlated with recurrence-free survival and immune infiltration

**DOI:** 10.1038/s41419-020-02973-1

**Published:** 2020-09-19

**Authors:** Xingbo Long, Huimin Hou, Xuan Wang, Shengjie Liu, Tongxiang Diao, Shicong Lai, Maolin Hu, Shengqi Zhang, Ming Liu, Hong Zhang

**Affiliations:** 1 Department of Urology, Beijing Hospital, National Center of Gerontology, No 1, Dongdan Dahua Road, Dongcheng District, Beijing, 100730 China; 2 Graduate School of Chinese Academy of Medical Science and Peking Union Medical College, No. 9, Dongdan Sanjiao Alley, Dongcheng District, Beijing, 100001 China; 3 Peking University Fifth School of Clinical Medicine, No 1, Dongdan Dahua Road, Dongcheng District, Beijing, 100730 China; 4 Department of Orthopaedic, Beijing Hospital, National Center of Gerontology, No 1, Dongdan Dahua Road, Dongcheng District, Beijing, 100730 China; 5 Institute of Cardiovascular Sciences and Key Laboratory of Molecular Cardiovascular Sciences, Peking University Health Science Center, Beijing, 100191 China

**Keywords:** Cancer microenvironment, Prognostic markers

## Abstract

Androgen deprivation therapy (ADT) is a cornerstone treatment for locally advanced or metastatic prostate cancer (PCa). However, its potential effects on the tumor immune microenvironment (TIM) of PCa patients and the underlying mechanism remain largely unclear. To explore the effects of ADT on PCa TIM, RNA sequencing was performed on six paired pre-ADT biopsy and post-ADT PCa lesions, and five paired paracancerous benign tissues from patients receiving neoadjuvant ADT with locally advanced PCa. Bioinformatics methods including ESTIMATE and ssGSEA were used to evaluate the stromal immune score and immune cell infiltration in PCa and paracancerous tissues. Weighted correlation network analysis was used to screen hub genes in the ADT-induced immune remodeling process. The results showed differences exist between PCa and paracancerous tissues in response to ADT. Compared with paracancerous tissues, the immune remodeling effect of ADT in PCa was more intense. *ZFP36*, *JUNB*, and *SOCS3* served as hub genes in the ADT-induced immune remodeling process and were associated with PSA recurrent-free survival in the TCGA and our neoadjuvant ADT cohort. To investigate the joint action of the above three hub genes, an immune signature score was constructed. The results showed that immune signature score-based immune subtypes reveal the heterogeneity of the immune microenvironment of PCa and showed significant differences in patient prognosis, tumor immune infiltration, mutation burden, and landscape.

## Introduction

Prostate cancer (PCa) is the most common cancer among men^1^. PCa progression is initially driven by abnormal activation of androgens and androgen-related signaling pathways. As such, androgen deprivation therapy (ADT) is a cornerstone for locally advanced or metastatic PCa treatment. ADT promotes the apoptosis of hormone-sensitive prostate epithelial cells, which leads to the involution of PCa^2^. Although the mechanisms of the direct antitumor effects of ADT have been widely studied, the potential profound effects on the PCa tumor immune microenvironment (TIM) remain largely unclear. Many standard-of-care therapies, including chemotherapy, radiotherapy, and small molecule inhibitor therapy, in addition to their on-target antitumor effects, have also demonstrated the ability to induce immunogenic modulation by altering the expression of proteins implicated in immune recognition and/or antigen processing in various cancers^3,4^. Recently, several in vivo and in vitro studies have shown that ADT could induce a complex immune cell infiltrate and increase the sensitivity of tumor cells to immune-mediated lysis and killing. More importantly, mice receiving a combination enzalutamide treatment with cancer vaccine had significantly increased overall survival compared to mice receiving no treatment or either monotherapy alone^5–7^. In addition, several clinical trials have revealed that combining ADT with specific checkpoint inhibitors or immunotherapy may potentially increase the antitumor effectiveness of immunotherapies^8–10^. These results suggest that ADT may also have indirect immunostimulatory effects. However, few studies have explored the potential immune remodeling effects of ADT in human PCa tissues and the underlying mechanism remains largely unknown.

The recent success of immune checkpoint inhibitors in cancers has led to renewed interest in tumor immunotyping, which help us to identify prognostic and guide the clinical individualized treatment. Several investigations have attempted to define a pan-cancer immune landscape ranging from broad classifications as immunologically cold or hot to six molecular subtypes^11^. However, such comprehensive classification of the TIM in PCa is currently unavailable.

PCa shows high genomic variability among different ethnic populations. Compared with Western populations, Chinese patients have a high frequency of CHD1 deletion with a relatively high percentage of mutations in androgen receptor upstream activator genes and a low rate of TMPRSS2-ERG fusion. This leads to highly variable clinical features, treatment response, and outcomes^12^. Currently, most studies regarding ADT are based on Western populations and the influence of ADT on Chinese PCa and paracancerous benign tissues remains incompletely characterized.

In this study, we performed quantitative transcriptome profiling of PCa and paracancerous benign tissues from patients prior to and following ADT using next-generation sequencing (RNA sequencing (RNA-seq)), and then analyzed and compared our data with public PCa databases to determine the immune signature behind ADT-induced immune remodeling and finally immunotyping of PCa patients according to the immune signature. We hope to determine whether ADT remodels the PCa TIM in the Chinese population and reveal the key immunologic and transcriptomic changes in PCa.

## Result

### The transcriptional landscape of PCa and paracancerous benign tissue responses to ADT

RNA-seq was performed on six paired pre- and post-ADT PCa lesions and five paired paracancerous benign tissues from patients with locally advanced PCa (Supplementary Table S1). First, we performed dimension reduction on these samples using principal component analysis (PCA) (Supplementary Fig. 1A). The results showed that PCa and paracancerous benign samples before ADT, PCa samples before and after ADT, and paracancerous benign samples before and after ADT can be clearly separated in two major dimensions (principal components 1 and 2); however, there are no clear boundaries between PCa and paracancerous benign samples after ADT (Supplementary Fig. 1A).

**Fig. 1:**
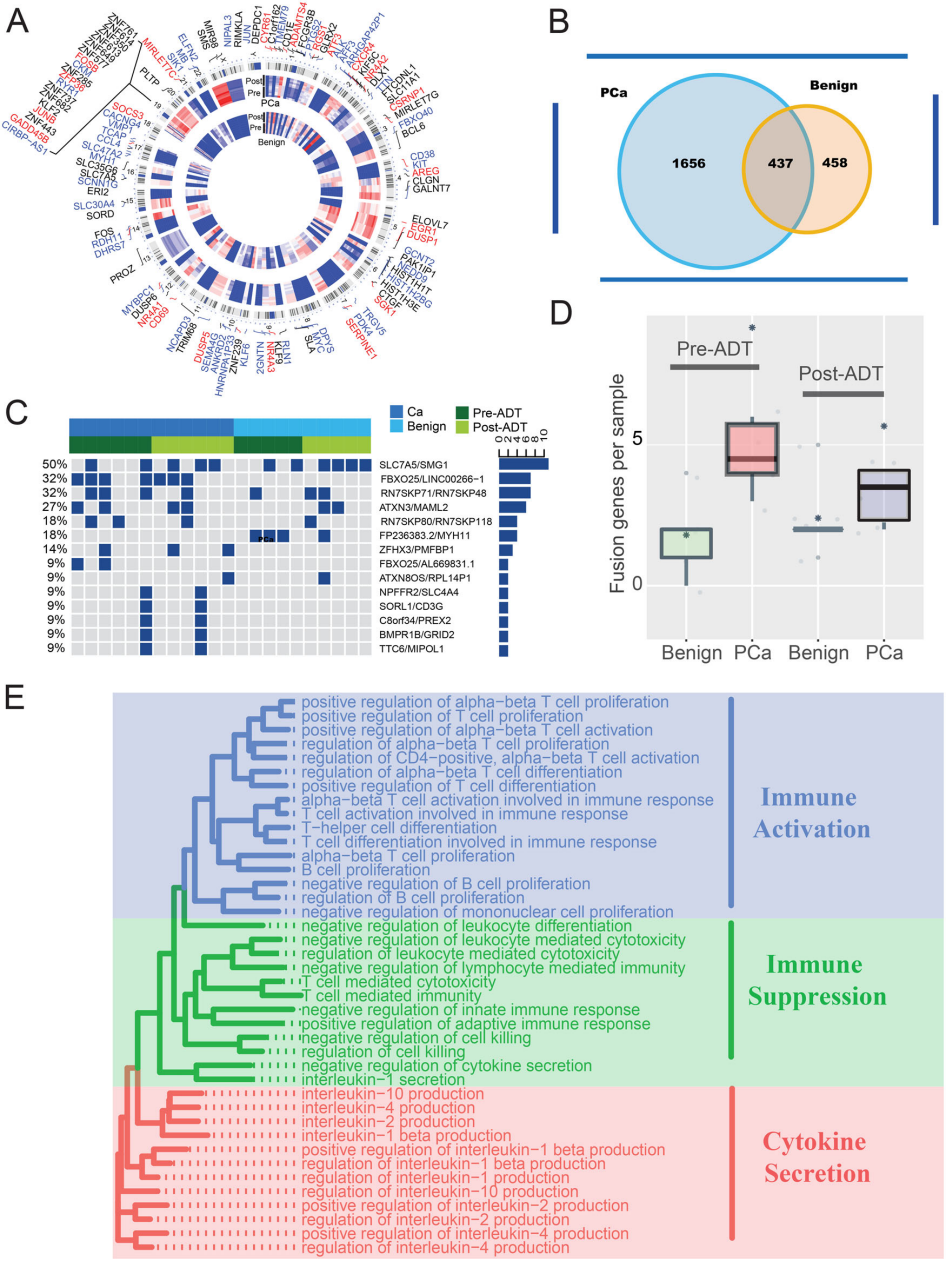
The transcriptional landscape of PCa and paracancerous benign tissues response to ADT. **a** Circular visualization of chromosomal positions for top DEGs 70 genes (top 35 upregulated and 35 downregulated genes) in our PCa and precancerous tissues response to ADT (innermost group: precancerous; outermost group: PCa). Black genes: DEGs in PCa tissues; blue genes: DEGs in precancerous tissues; red genes: represent co-DEGs in both tissues. **b** Venny diagram of DEGs in PCa and precancerous tissues. **c** Top 14 most frequency fusion genes in PCa and precancerous tissues pre- or post-ADT. **d** The box plot shows the number of fusion genes in different groups. **e** Representative enriched GO functions of DEGs in PCa tissues response to ADT. GO categories are grouped according to functional theme.

Then, we identified a total of 2093 differentially expressed genes (DEGs) (1440 upregulated and 653 downregulated; Supplementary Table S2 and Fig. 1a) at least twofold (false discovery rate < 0.05) in PCa samples in response to ADT. Similarly, we identified a total of 895 DEGs (449 upregulated and 446 downregulated; Supplementary Table S2 and Fig. 1a) in paracancerous benign samples in response to ADT. Compared with PCa data, we found that PCa samples had more DEGs and they shared 48.8% of DEGs (Fig. 1b).

Genomic rearrangements have been hypothesized to be a mechanism driving prostate carcinogenesis^13,14^. In our study, a total of 59 genomic rearrangements were detected (Supplementary Table S3). We identified a mean of five rearrangements per sample and PCa samples showed high rearrangements compared to the paracancerous benign samples (*P* = 0.016). However, unlike the Western population-based study, many fusion genes could not be detected after ADT^15^ and there was no difference between samples pre- and post-ADT (*P* = 0.16) (Fig. 1c, d). Moreover, few ERG family gene fusions in our cohort were detected, which is consistent with previous studies based on the Chinese population^12,16^.

To identify biological pathways perturbed following ADT in PCa samples, enrichment analyses were performed. Gene Ontology (GO) function analysis showed that DEGs were enriched in both immune activation and immune suppression functions (Fig. 1e and Supplementary Table S4). Similarly, many immune-related and proliferation-related pathways were enriched by using gene set enrichment analysis (GSEA) Kyoto Encyclopedia of Genes and Genomes (KEGG) pathway analyses (Supplementary Table S4 and Supplementary Fig. 1B). Consistent with a previous study, the WNT signaling pathway was also enriched (Supplementary Fig. 1B)^15^. Regarding paracancerous benign samples apart from immune-related functions and pathways, many skeletal muscle-related pathways were also enriched (Supplementary Fig. 1C, D and Supplementary Table S4).

The above results indicated that ADT treatment may activate the PCa TIM, and that both immune activation and immune suppression functions have been stimulated.

### ADT remodel PCa TIM

To further confirm the effect of ADT on the TIM of PCa, several bioinformatics methods, including Estimation of STromal and Immune cells in MAlignant Tumor tissues using Expression data (ESTIMATE)^17^ and single-sample GSEA (ssGSEA)^18^ methods, were used to evaluate the PCa stromal immune score and immune cell infiltration in samples before and after ADT.

**Fig. 2:**
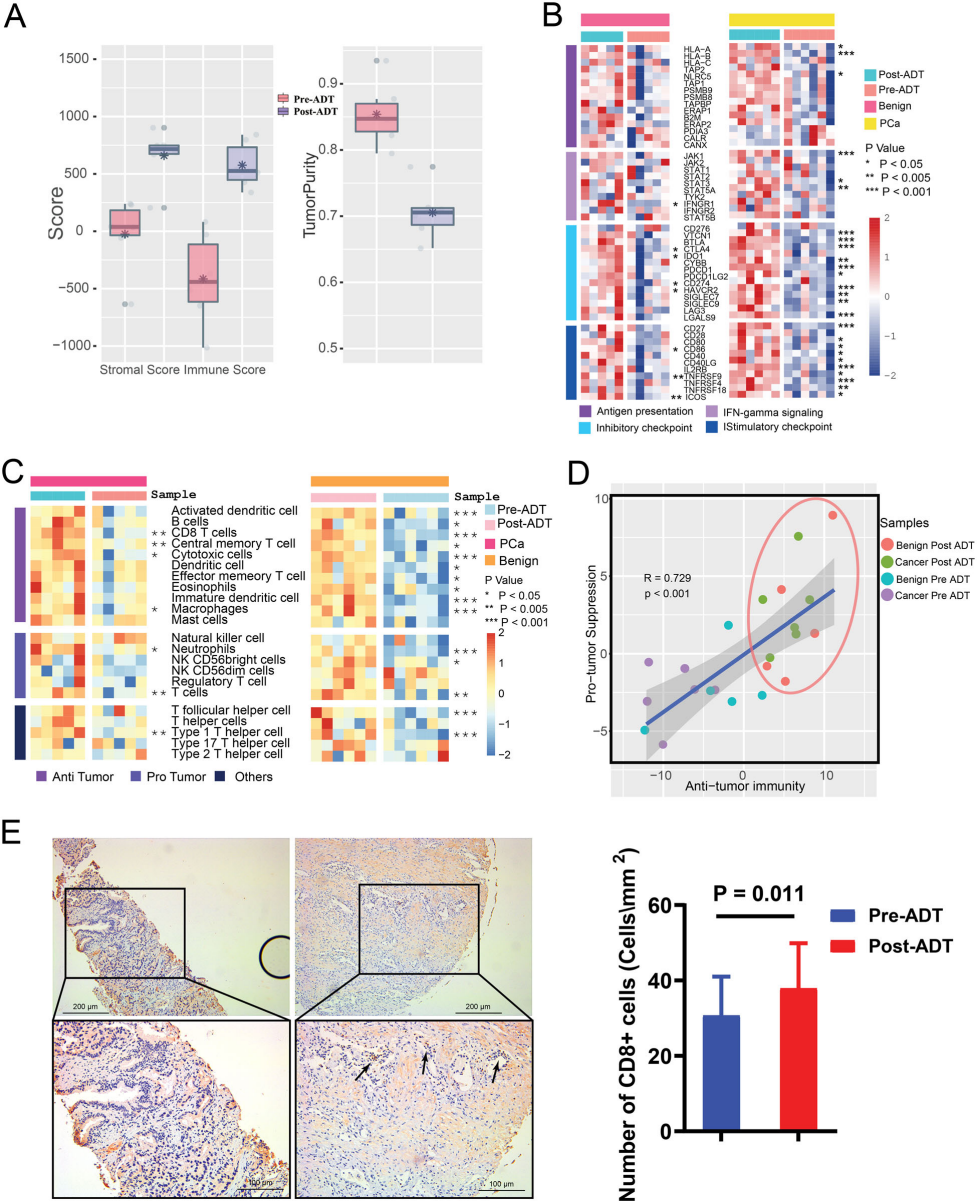
ADT remodel PCa TIM. **a** Stromal and immune score and tumor purity calculated by ESTIMATE method in PCa and precancerous tissues pre- and post-ADT. **b** Expression of immune-related genes and immune checkpoint genes in PCa and paracancerous benign tissues pre- and post-ADT. **c** ssGSEA analysis identifying the relative infiltration of immune cell populations in PCa and precancerous tissues pre- and post-ADT. The relative infiltration of each cell type is normalized into a *z* score. **d** Correlation between infiltration of cell types executing antitumor immunity (activated dendritic cell, CD8 T cell, central memory T cell, cytotoxic cell, effector memory T cell, nature killer cell, NK CD56 bright cell, T cell, T-helper cell, Type 1 T-helper cell, and Type 17 T-helper cell) and cell types executing protumor, immune suppressive functions (immature dendritic cell, macrophages neutrophils, NK CD56 dim cell, regulatory T cell, and Type 2 T-helper cell) in PCa and precancerous tissues pre- and post-ADT. R coefficient of Pearson’s correlation. The shaded area represents 95% confidence interval. **e** Left: the expression of CD8 in samples with (43 patients) and without (22 patients) ADT (original magnification ×200).

The ESTIMATE analysis showed that the immune score and stromal score were significantly elevated, while tumor purity was significantly lower after ADT (Fig. 2a). In addition, the expression of many antigen presentation, interferon-γ (IFN-γ) signaling and immune checkpoint genes was elevated after ADT. This elevation was more dramatic in PCa samples than that in the paracancerous samples (Fig. 2b). ssGSEA analysis revealed the infiltration level of 22 immune cell types in the immune microenvironment. We classified immune cell types into three categories as follows: (1) cells executing antitumor reactivity; (2) cells delivering protumor and mediating immunosuppression; and (3) others. Protumor scores and antitumor immunity scores (sum of antitumor and protumor cells normalized ssGSEA scores) were generated. The results showed that the infiltration level of many immune cells was increased after ADT and these changes were more dramatic in PCa samples (Fig. 2c). Both anti- and protumor immune cells were increased in almost all samples after ADT (Fig. 2d) and Pearson’s correlation analysis showed that protumor and antitumor immunity scores were positively associated (Fig. 2d).

According to the ssGSEA analysis, we found that the infiltration level of CD8+ T cells was dramatically increased after ADT. Immunohistochemical assays further confirmed that CD8+ T cells were abundant in PCa TIM compared with samples without ADT (Fig. 2e).

All of the above results implicated that ADT could significantly change PCa TIM. Compared with paracancerous tissues, the immune remodeling effect of ADT in PCa was more intense.

### Screening hub genes in the ADT-induced immune remodeling process using weighted gene co-expression network analysis (WGCNA)

**Fig. 3:**
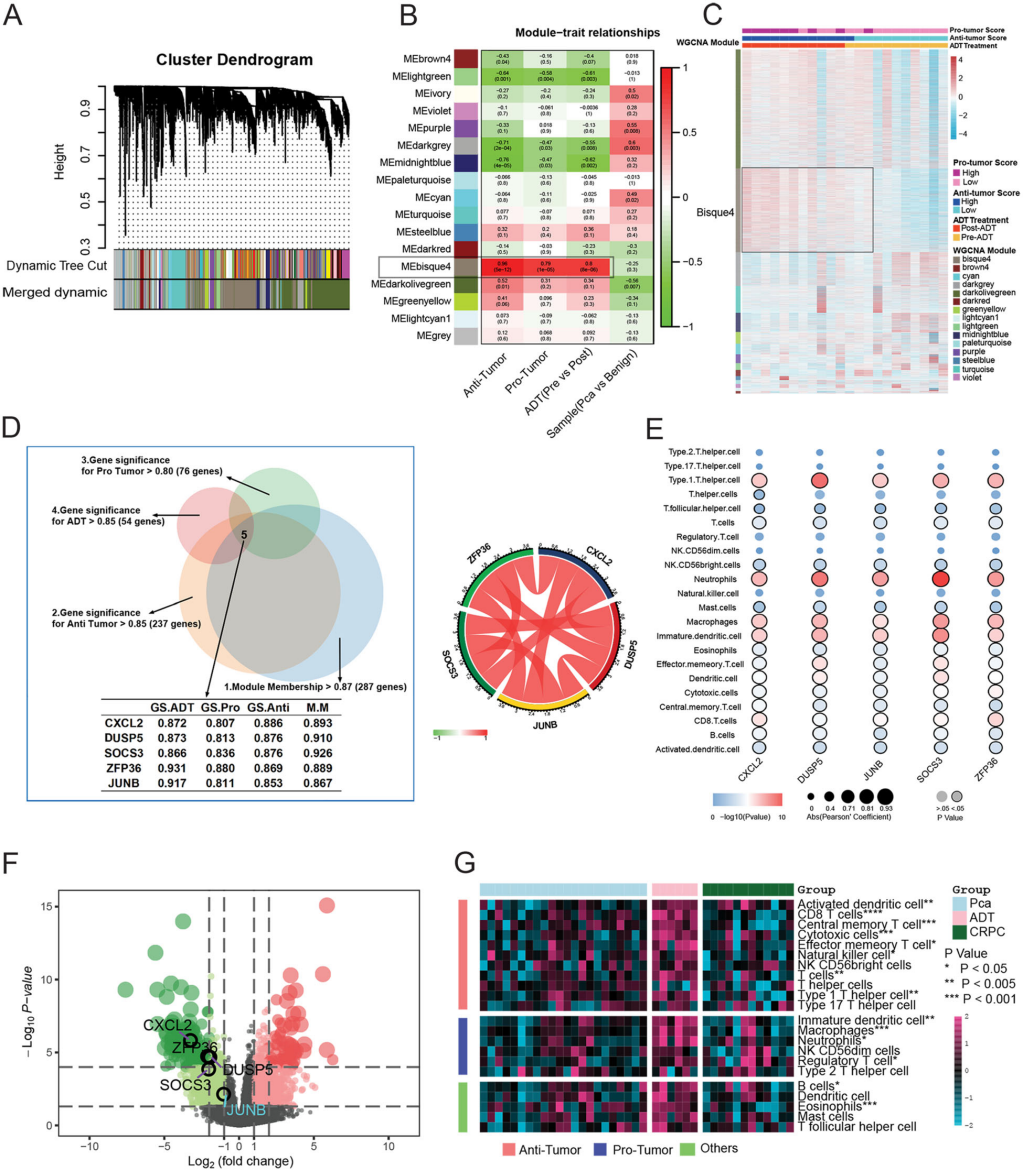
Screening hub genes in ADT-induced immune remodeling process using WGCNA. **a** Dendrogram generated using the WGCNA and identified 17 distinct gene modules. Each module is assigned by a unique color. **b** Pearson’s correlation coefficient (PCC) matrix between module eigengenes (MEs) and clinical traits. The PCC values range from −1 to 1 depending on the strength of the relationship. A positive value indicates that the genes within a particular co-expression module increase as the clinical trait increases, whereas the opposite is true if the PCC is negative. Each PCC value is accompanied by the corresponding *P*-value in brackets. **c** WGCNA clustering of differentially expressed genes. **d** Left: Venny diagram shows the conditions for screening hub genes. Right: Pearson’s correlation coefficient of five hub genes. **e** Pearson’s correlation coefficient between 5 hub genes and the infiltration level of 22 immune cell types in the PCa TIM. **e** Volcano Plot visualizing the DERs between primary PCa and CRPC samples, which was screened by DESeq2. The colorized points in scatter plot represent the DEGs with statistical significance (FDR < 0.05, |log2FC| > 1). Five hub genes were significantly downregulated in CRPC samples. **f** The infiltration level of 22 immune cell types in the primary PCa, ADT, and CRPC samples.

To further explore the hub genes in the ADT-induced immune remodeling process, WGCNA using the top 8269 variation genes in 22 samples was used to compile the co-expression network. Keeping to the scale-free topology criterion, *β* = 16 was considered in this study. Following dynamic tree cutting, the topological overlap clustering dendrogram identified 17 distinct gene modules (Fig. 3a). The gray module consisted of genes that did not group into any specific module. To identify co-expression modules associated with sample traits (protumor immunity scores, antitumor immunity scores, pre- vs. post-ADT, and PCa vs. paracancerous), we assessed the relationship of the above four sample traits with the module eigengene. Figure 3b, c show that the bisque4 module has the strongest association with antitumor immunity (0.96, *P* < 0.001), protumor immunity scores (0.79, *P* < 0.001), and ADT (0.80, *P* < 0.001).

Therefore, we focus on the bisque4 module. Not surprisingly, GO and KEGG enrichment analyses showed that genes in the bisque4 module were enriched in many immune-related functions and pathways (Supplementary Fig. 2A, B). Then, we identified hub genes in the bisque4 module based on four scores as follows: (1) Module Membership > 0.87 (high connectivity genes in the module); (2) gene significance for antitumor immunity scores > 0.85; (3) gene significance for protumor immunity scores > 0.80; and (4) gene significance for ADT > 0.85. After screening, five highly correlated hub genes [*CXCL2* (*C-X-C motif chemokine ligand 2*), *DUSP5* (*dual specificity phosphatase 5*), *SOCS3* (*suppressor of cytokine signaling 3*), *ZFP36* (*ZFP36* *ring finger protein*), and *JUNB* (*JunB* *proto-oncogene*)] remained (Fig. 3d). Pearson’s correlation analysis further confirmed that five hub genes were highly correlated with the majority of the infiltration level of 22 immune cell types in the immune microenvironment (Fig. 3e).

Then, we validated these five hub genes in the The Cancer Genome Atlas (TCGA) PCa cohort. The results showed that these five hub genes showed similar expression correlations (Supplementary Fig. 2C) and correlated with many immune cell types (Supplementary Fig. 2D). In the other castration-resistant PCa (CRPC) cohort containing primary PCa and CRPC samples, differential gene expression analysis showed that these five genes were all significantly downregulated in CRPC samples compared with primary PCa samples (Fig. 3f). Then, we combined our data with the above CRPC data and compared the infiltration levels of 22 immune cell types in TIM primary PCa, ADT, and CRPC samples. After removing the batch effect, the results showed that the infiltration levels of many immune cell types were increased in ADT samples compared to the primary PCa samples but then decreased in CRPC samples.

### ZFP36, JUNB, and SOCS3 and their immune signature scores were associated with patients’ prostate specific antigen (PSA) RFS

**Fig. 4:**
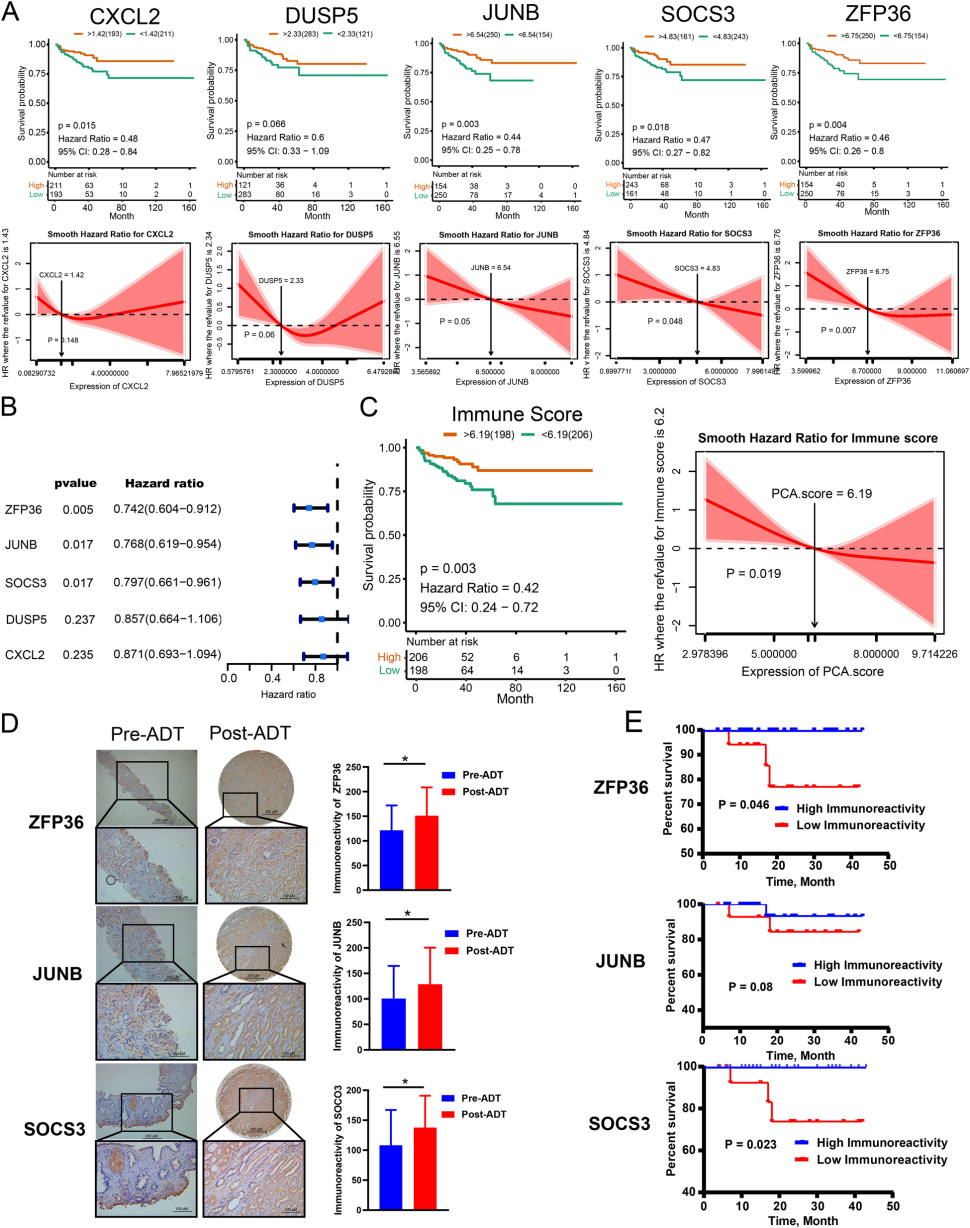
*ZFP36*, *JUNB*, and *SOCS3* and immune signature score constructed by them were associated with the PSA RFS in TCGA and our neoadjuvant ADT cohort. **a** Kaplan–Meier curves (upper) and smooth HR curves (down) showed with optimal cutoff values; high level of hub genes presented a favorable PSA PRS in TCGA cohort. **b** Univariate Cox regression analysis showed that *ZFP36*, *JUNB*, and *SOCS3* expression affects patient PSA RFS. **c** Kaplan–Meier curves (left) and smooth HR (right) analysis confirmed the high immune signature score show favorable PSA RFS. **d** Immunohistochemical assay of *ZFP36*, *JUNB*, and *SOCS3* in pre-ADT biopsy and post-ADT radical prostatectomy samples of our neoadjuvant ADT cohort (×200). **e** Kaplan–Meier curves show high level of *ZFP36*, *JUNB*, and *SOCS3* scores in radical prostatectomy samples presented a favorable PSA PRS in our neoadjuvant ADT cohort.

Previous studies showed that patients with an activated immune microenvironment showed favorable clinical outcomes in various cancers. To evaluate the prognostic value of five hub genes as a linear variable, we first performed Kaplan–Meier curves and smooth hazard ratio (HR) curves of PSA recurrence-free survival (RFS) in the TCGA PCa cohort. Kaplan–Meier curves showed that under the optimal cutoff point, the high expression groups had higher PSA RFS than the low expression groups (Fig. 4a). The smooth HR curves of PSA RFS further confirm that the HR of PSA RFS decreased with increasing gene expression levels in five hub genes, referring to their corresponding cutoff values and vice versa.

Then, univariate Cox regression analysis was used to further determine the correlation between five hub genes and patients’ PSA RFS. As expected, the hub genes *ZFP36* (*P* = 0.005, HR = 0.74), *JUNB* (*P* = 0.017, HR = 0.77), and *SOCS3* (*P* = 0.017, HR = 0.80) were significantly associated with PSA RFS and their high expression could result in a favorable prognosis for patients (Fig. 4b).

Given the high correlation and significant impact on PSA RFS, a PCA-based variable, the immune signature score was calculated to compress the expression level of three hub genes (*ZFP36*, *JUNB*, and *SOCS3*). Kaplan–Meier curves, smooth HR analysis, and univariate Cox regression analysis (*P* = 0.008, HR = 0.75, 95% confidence interval HR = 0.61 ~ 0.93) all confirmed the correlation between the immune signature score and PSA RFS (Fig. 4c). Under the optimal cutoff point according to the Kaplan–Meier curves, we separated the TCGA cohort into two subtypes: a 198-sample immune-high subtype with relatively high expression of immune signature score and a 206-sample immune-low subtype with low immune signature score. Multivariate Cox analysis showed that immune signature score-based immune subtype, pathology T stage, and Gleason sum score were presented as independent predictors for PSA RFS in the TCGA cohort (Supplementary Table S5).

Then, we validated the above results in our neoadjuvant ADT cohort. Immunohistochemical analysis showed that the immunoreactivity of *ZFP36*, *JUNB*, and *SOCS3* was significantly higher after ADT in our neoadjuvant ADT cohort (Fig. 4d). Kaplan–Meier curve analysis showed that high immunohistochemical activity levels of *ZFP36*, *JUNB*, and *SOCS3* in radical prostatectomy PCa tissues were associated with favorable PSA RFS in our neoadjuvant ADT cohort (Fig. 4e).

### Immune signature score-based subtypes were associated with immune infiltration in the TCGA and the international cancer genome consortium (ICGC) cohorts

**Fig. 5:**
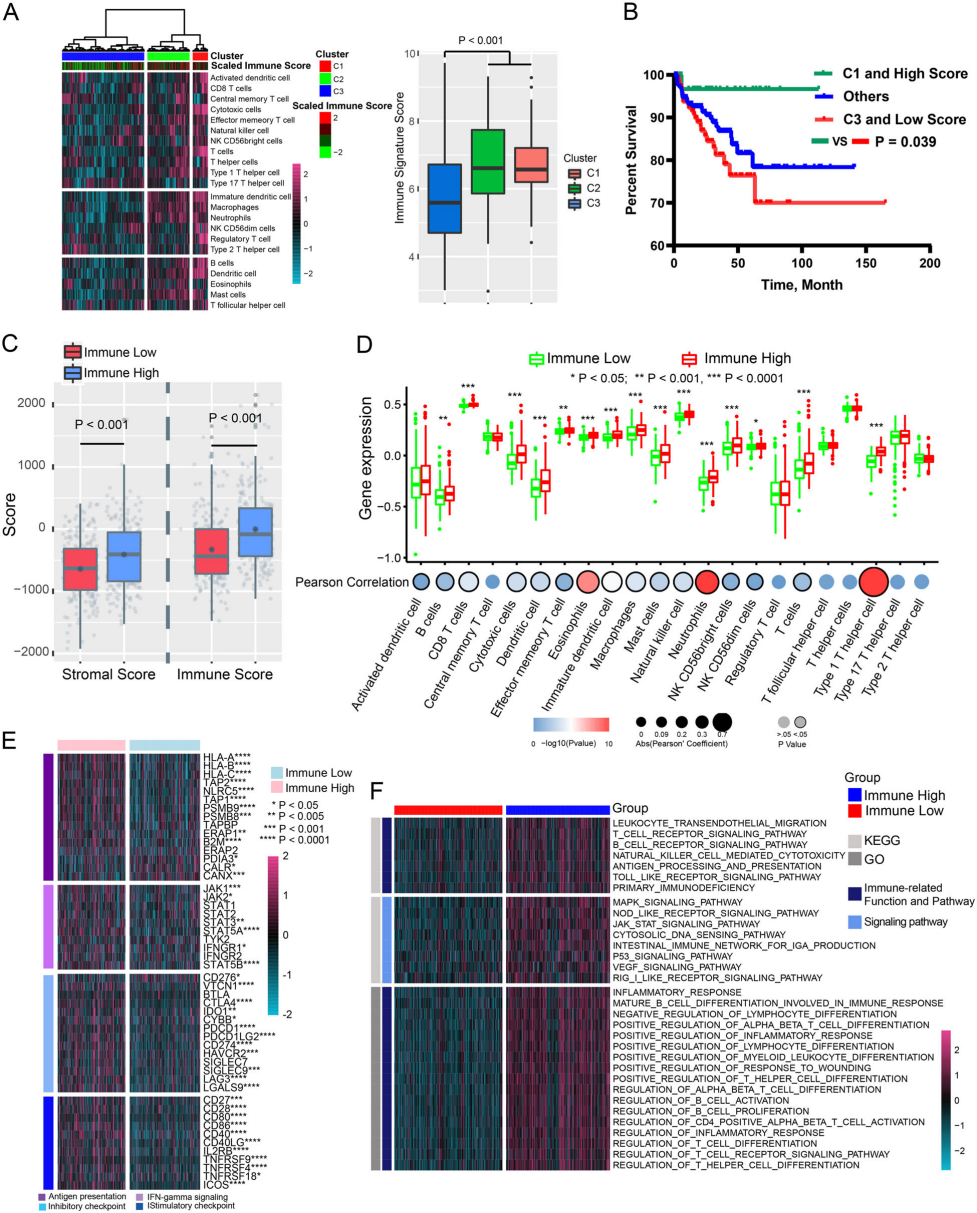
Immune signature score-based subtypes was associated with the immune infiltration in TCGA cohort. **a** Left: heatmap of 22 related cell types across 404 PCa samples distinguished three immunological patterns in TCGA cohort. Right: the box plot shows immune signature score between the three immunological patterns in TCGA cohort. **b** Kaplan–Meier curve of PSA RFS in different subgroups. **c** Stromal and immune score calculated by ESTIMATE in immune-high and -low subtypes. **d** Difference of infiltration level of 22 immune cell types between immune-high and -low subtype and the correlation of infiltration level of 22 immune cell types and immune signature score. **e** Expression of immune-related genes and immune checkpoint genes in immune-high and -low subtypes. **f** Heatmap of the differentially enriched KEGG pathways and Go functions calculated by gene set variation analysis analysis between immune-high and -low subtypes.

We next characterized the immunologic profiling and molecular differences between the immune signature score-based subtypes in the TCGA and ICGC cohorts. First, supervised clustering using 22 immune-related cell-type scores was applied to all TCGA and ICGC samples, and 3 distinct subtypes were revealed in the TCGA (Fig. 5a left) and ICGC (Supplementary Fig. 3A left) cohorts. Specifically, subtypes C1, C2, and C3 exhibited high, middle, and low enrichment levels for 22 immune cell infiltration levels, respectively. Subtypes C1 and C2 had significantly higher immune signature scores than C3 (TCGA cohort: Fig. 5 right.; ICGC: Supplementary Fig. 3A right) and presented high enrichment for samples classified into the immune-high subtype, whereas subtype C3 was enriched for samples belonging to the immune-low subtype. Then, we further classified patients into three groups as follows: Group 1—samples belong to both C1 and immune-high subtype; group 2—samples belong to C3 and immune-low subtype; group 3—others. Kaplan–Meier analysis showed that group 1 had significantly favorable PSA RFS, whereas group 2 had the worst RFS (Fig. 5b).

ESTIMATE analysis further confirmed that the immune-high subtype had a significantly higher immune sore in both the TCGA (Fig. 5c) and ICGC cohorts (Supplementary Fig. 3B). Moreover, the infiltration levels of many immune cell types were significantly elevated in the immune-high subtype compared with the immune-low subtype, and the immune signature score was significantly correlated with those immune cells in both the TCGA (Fig. 5d) and ICGC cohorts (Supplementary Fig. 3C). In addition, the expression of many immune, antigen presentation, IFN-γ signaling-related and immune checkpoint genes was elevated in the immune-high subtype in both the TCGA and ICGC cohorts (Fig. 5e and Supplementary Fig. 3D).

Gene set variation analysis enrichment analysis showed that many immune-related KEGG pathways and GO functions were enriched in the immune-high subtype in the TCGA (Fig. 5f) and ICGC cohorts (Supplementary Fig. 3E, F).

### Difference in somatic mutation landscape between immune-high and -low subtypes

**Fig. 6:**
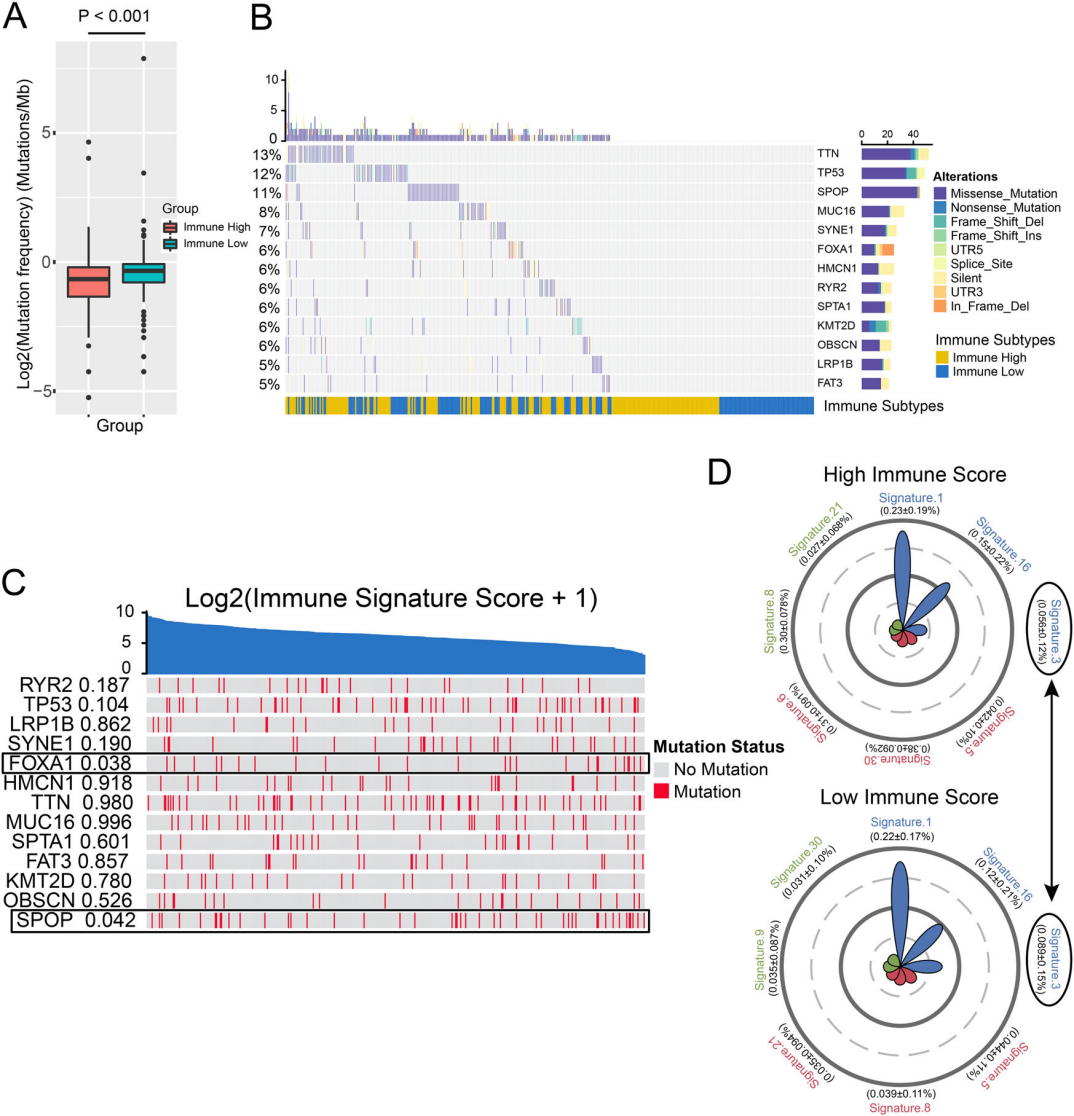
Difference of somatic mutation landscape between immune-high and -low subtype. **a**. Tumor mutation burden between two immune subtypes. **b** The somatic mutation landscape of high mutation frequency genes. **c** Correlations of high mutation frequency genes and immune signature score. **d** Radar chart showed the top seven mutational signatures in immune-high (up) and -low (low) subtypes. Signature 3 contribute differently in immune-high and -low subtypes.

To investigate whether differences exist in the somatic mutation frequencies between the two subtypes, we first found that the immune-low subtype presented a significantly higher tumor mutation burden than the immune-high subtype (Fig. 6a). Then, we filtered genes with a mutation rate >5% and identified 13 genes (Fig. 6b). *TTN*, *TP53*, and *SPOP* were the three genes with the highest mutation rates in the TCGA cohort. Among them, *SPOP* and *FOXA1* mutations were correlated with the immune-low subtype (Fig. 6c). Mutations in *SPOP* and *FOXA1* have been proven to be important events in the development and drug resistance of PCa^19,20^. Finally, we compared the mutational signatures^21^ between two immune subtypes. Multivariate analysis of variance was used to analyze the association of mutational signatures with two subtypes. We identified a significant difference between the two subtypes (Fig. 6d). Signature 3 related to DNA double-strand break repair was higher in the immune-low subtype than in the immune-high subtype (Fig. 6d).

## Discussion

PCa is generally an indolent and “cold” tumor with a relatively low tumor mutation burden and minimal T-cell and immune infiltrates^22^. Many immunotherapeutics, such as PD-1 inhibitors, show promise in many cancers^23^. It is unlikely that any of the immunotherapeutics alone can dramatically change PCa outcomes^22,23^. Therefore, many investigators have attempted to integrate immunotherapy into the existing standard treatments, such as ADT, radiotherapy, or chemotherapy, to improve the efficacy of immunotherapy and patients’ overall survival in tumor-bearing hosts. Previous researchers showed that ADT affects the immune system by inducing thymic regeneration, leading to increased production of naive T cells^24^, decreasing CD4+ T-cell tolerance^25^, and increasing CD4+ effector T cells^26^. Given the potential immune effects of ADT, multiple clinical trials have been performed to evaluate the synergistic effect of ADT and immunotherapy^5,6,8–10,27^, and some of them have shown promising results. Unfortunately, few studies have comprehensively evaluated the effect of ADT therapy on TIM in PCa.

In our study, we found that ADT could significantly increase the infiltration and activity of immune cells in PCa TIM. A variety of immune-related genes and pathways, including antigen presentation, immune checkpoints (*PD-1*, *PD-L1*, *CTLA-4*, etc.), and the IFN-γ signaling pathway were significantly activated after ADT treatment. Further analysis revealed that antitumor immunity- and protumor immunity (immunosuppression and immune escape, etc.)-related inflammatory cell activity and pathways were significantly activated after ADT treatment. More importantly, the effect of ADT on the activation of the immune microenvironment in tumor tissues is more significant than that in adjacent normal tissues.

Through the analysis of primary advanced PCa, neoadjuvant ADT, and CRPC samples, we found that the activation of the immune microenvironment caused by ADT may be time dependent. Early or short-term ADT treatment can significantly activate the immune microenvironment of PCa, and as ADT treatment continues or enters the CRPC phase, the immune microenvironment activation caused by ADT treatment becomes very limited. This suggests that combined immunotherapy with early-stage ADT therapy may be more effective in increasing the efficacy of immunotherapy.

At present, there have been many attempts to conduct molecular classification of PCa, and further study of the heterogeneity of PCa will provide more in-depth understanding and better personalized treatment for PCa patients^28^. Unfortunately, as the immune infiltration of PCa is not obvious, few studies have conducted molecular typing of PCa based on immune infiltration or immune-related genes. Interestingly, we found that the immune signature score composed of *SOCS3*, *JUNB*, and *ZFP36*, the hub genes driven by the ADT-induced PCa immune remodeling process, was significantly correlated with the immune infiltration of PCa and PSA RFS. Based on the immune signature score, we divided the patients into two subtypes with high and low immune scores. The two subtypes showed significant differences in patient prognosis, tumor immune infiltration and mutation landscape. This suggests that two immune subtype differences are essential and reflect the heterogeneity of the immune microenvironment of PCa, which is worthy of further study.

The expression of *SOCS3*, *JUNB*, and *ZFP36* is highly correlated in PCa, and through immunohistochemical analysis we found *SOCS3*, *JUNB*, and *ZFP36* proteins were mainly expressed in PCa epithelial cells with a small amount in tumor stroma. The expression of these genes is closely related to the occurrence and development of PCa^29,30^. *SOCS3* is a cytokine-inducible negative regulator of cytokine signaling. The expression of this gene is induced by various cytokines, including interleukin (IL)-6, IL10, and IFN-γ. The protein encoded by *SOCS3* can bind to JAK2 kinase and inhibit the activity of JAK2 kinase^31^. *JUNB* is a close homolog of c-Jun with tumor suppressive function in the myeloid lineage^29^, and *ZFP36* is a CCCH zinc finger-containing protein that destabilizes mRNA by binding to an AU-rich element. *ZFP36* could negatively regulate nuclear factor-κB (NF-κB) signaling at the transcriptional corepressor level, by which it may regulate inflammatory gene transcription^32^. The three genes are key regulators of NF-κB, STAT3, and JNK signaling. Activation and inhibition of these pathways are associated with secretion of various cytokines and immune regulation. However, their effects on the immune microenvironment of PCa have been poorly studied. Our study found that *SOCS3*, *JUNB*, and *ZFP36* may play an important role in the ADT-induced immune microenvironment remodeling process of PCa. Their high expression is associated with patient prognosis and immune infiltration. The underlying mechanisms deserve further study.

In conclusion, ADT remodels the TIM in PCa. Immune-related genes *ZFP36*, *JUNB*, and *SOCS3* may play an important role in the ADT immune remodeling process, and PCa immunotyping based on the above three genes showed great differences in PSA RFS, immune infiltration, and mutation landscape in PCa.

## Materials and methods

### Study population

This study was approved by the ethics committee at Beijing Hospital (2018BJYYEC-085-03) and informed consent was obtained from all patients.

### Samples for RNA sequencing

Six paired pre- and post-ADT PCa lesions and five paired paracancerous benign tissues were obtained from patients receiving neoadjuvant ADT (bicalutamide 50 mg per day and goserelin and 3.6 mg every 4 weeks with 2 cycles) with locally advanced PCa. Pre-ADT tissues were obtained from prostate biopsy tissue before ADT, and by using the whole mount technique, we obtained post-ADT tissues from the corresponding place in the radical prostatectomy samples where the pre-ADT prostate biopsy tissues were obtained. In total, 22 samples were obtained for RNA-seq analysis. Detailed descriptions of RNA-seq and bioinformatics analyses are included in the Supplementary Materials and Methods. Patient demographic information is summarized in Supplementary Table S1 Part 1.

### Neoadjuvant ADT cohort for validation and PSA RFS analysis

Forty-three radical prostatectomy samples with primary advanced PCa receiving neoadjuvant ADT before radical prostatectomy and 22 corresponding prostate biopsy samples before ADT were retrospectively recruited in our study. Forty-three radical prostatectomy samples were used for PSA RFS analysis. Patients with confirmed metastasis, positive surgical margin, a performance-status score of 3 or more on the Eastern Cooperative Oncology Group scale and other histopathological types, except adenocarcinoma, were excluded from the study. Patients with simultaneous cancers other than PCa were also excluded. All samples were collected for immunohistochemistry. Patient demographic information is summarized in Supplementary Table S1 Part 2.

### TCGA PCa cohort

The TCGA cohort comprised 404 PCa patients’ RNA-seq raw count and fragments per kilobase million (fpkm) data were obtained from the TCGA provisional database. The selection criteria were as follows: (1) availability of PSA RFS data and mRNA expression data; (2) the tissue used for RNA-seq was frozen tissue and formalin-fixed paraffin-embedded tissue was excluded. A total of 404 patients were included in the study. Demographic information is summarized in Supplementary Table S1 Part 3.

### ICGC PCa cohort

A total of 144 patents with mRNA expression data (raw count and fpkm) were included in the ICGC PCa cohort (Prostate Adenocarcinoma–CA, https://dcc.icgc.org/projects/PRAD-CA).

### Castration-resistant prostate cancer data

The CRPC cohort containing 25 primary PCa and 12 CRPC samples and their paired-end RNA sequencing fastq raw data were obtained from the SRA database (SRP073789)^33^. The bioinformatics analysis process was the same as that used in our RNA-seq data (Supplementary Materials and Methods). When combining the analysis of those data with our data, we used Surrogate Variable Analysis methods (R package: SVA)^34^ to remove the batch effect between the above two data points.

### RNA extraction

RNA was extracted from formalin-fixed, paraffin-embedded tissue. After hematoxylin and eosin-stained slide review, tumor and paracancerous benign tissues were selected. We then manually microdissected the corresponding tissue from unstained, 5 μm-thick tissue sections (ten sections for RNA). For RNA-seq, we purified RNA using the RNeasy FFPE Kit from FFPE slides. RNA quality was determined with the DV_200_ value (DV_200_ > 30%) by a Caliper BioAnalyzer 2100 Instrument. RNA samples were submitted to WuXi NextCode for next-generation sequencing with TruSeq RNA Exome. Paired-end sequencing (2 × 150 bp reads) was performed on successful RNA libraries using the Illumina HiSeq X-Ten platform. During the experiment, investigators were blinded to patients information.

### Construction of immune signature score

To investigate the joint action of the immune-related genes *SOCS3*, *ZFP36*, and *JUNB*, an immune signature score was constructed. In the present study, we found that SOCS3, ZFP36, and JUNB influence patient PSA RFS and their expression levels were also confirmed to be highly associated, prompting us to focus on these three immune-related genes. We identified a comprehensive immune signature score to explain the original expression level of immune-related genes *SOCS3*, *ZFP36*, and *JUNB*, which was calculated by PCA. The new PCA-based variable immune signature score was derived from the first principal component that represented 91.8% and 92.8% of the variation in the TCGA and ICGC cohorts, respectively. The coefficients (normalized loading) of SOCS3, ZFP36, and JUNB to the first principal component are shown: JUNB (0.309), SOCS3 (0.354), and ZFP36 (0.337) for TCGA and JUNB (0.300), SOCS3 (0.368), and ZFP36 (0.334) for the ICGC cohort.

### Supervised hierarchical clustering

Supervised hierarchical clustering based on immune-related cell types (ssGSEA immune cell score) was performed using the hclust R function via Ward’s clustering and 1 − Pearson’s correlation distance. According to the hierarchical results, we divided the TCGA and ICGC cohorts into three clusters.

### Statistical analysis

All statistical tests were performed using R software 3.6.1. Data were expressed as mean ± SD. The number of samples for RNA-seq meets the requirement of biological repeats for RNA-seq bioinformatics analysis (≥3 samples each group). PCA is used for dimensionality reduction and estimate of variation within each group. The *χ*^2^-test or Fisher’s exact test was used for categorical data when appropriate and a two-sample Wilcoxon test (Mann–Whitney test) was used for continuous data. Log-rank test Kaplan–Meier curve, smooth HR curves^35^, and Cox regression for survival analysis were performed by R package “survival.” and “smoothHR”. The survival of patients belonging to different defined groups was compared using the Kaplan–Meier method, with the *p*-value determined by the log-rank (Mantel–Cox) test. Pearson’s correlation was used to evaluate the correlation between two objects. For all statistical analyses, *P* < 0.05 was considered statistically significant. Statistical tests for every figure are justified as appropriate and all data meet the assumptions of the responding tests.

Bioinformatics processes and materials and details relating to tissue immunohistochemistry are described in the Supplementary Materials and Methods.

## Supplementary information

Supplementary Materials and Methods

Supplementary Figure Legends

Supplementary Table Legends

Supplementary Figure 1

Supplementary Figure 2

Supplementary Figure 3

Supplementary Table S1

Supplementary Table S2

Supplementary Table S3

Supplementary Table S4

Supplementary Table S5

## Data Availability

The Raw data of RNA sequencing of our ADT patients in this manuscript have been deposited in the National Center for Biotechnology Information Gene Expression Omnibus DataSets under accession number GSE150368. The RNA-seq raw count and fpkm data of TCGA and ICGC cohort were obtained from the TCGA provisional database and ICGC PCa cohort (Prostate Adenocarcinoma–CA, https://dcc.icgc.org/projects/PRAD-CA), respectively. Sequencing fastq raw data of CRPC patients was obtained from the SRA database (SRP073789).
