# Supplementary material for: Immune signature driven by ADT-induced immune microenvironment remodeling in prostate cancer is correlated with recurrence-free survival and immune infiltration
Source: Cell Death Dis. 2020 Sep 19;11(9):779. doi: 10.1038/s41419-020-02973-1 (PMC7502080; doi:10.1038/s41419-020-02973-1)
Supplement: Supplementary file 1 — Supplementary Materials and Methods [file 41419_2020_2973_MOESM1_ESM.docx]

**Supplementary Materials and Methods**

*Bioinformatics*

The quality of raw reads was first assessed using FastQC. After filtering out low-quality bases and adaptors using Trimmomatic-0.36 (<http://www.usadellab.org/cms/?page=trimmomatic>), reads were mapped to the human genome assembly GRCh38 (ftp://ftp.ensembl.org/pub/release-90/fasta/homo_sapiens/Homo_sapiens.GRCh38.dna.primary_assembly.fa.gz) using HISAT2^1^. Samples were subjected to quality control by examining the percentage of reads uniquely mapping to the genome, the percentage of reads mapping to known protein coding sequences, and the number of genes with 90% base coverage.

Gene fusions were identified by mapping reads to the human genome using STAR-Fusion version v1.6.0^2^. Differentially expressed genes (DEGs) were identified by counting the number of reads mapping to each gene from Ensemble 90 (ftp://ftp.ensembl.org/pub/release-90/fasta/homo_sapiens/Homo_sapiens.GRCh38.dna.primary_assembly.fa.gz) using Htseq-count^3^. Transcripts per million (TPM) were analyzed using Stringtie^4^ software. The R package DESeq2^5^ was used to detect DEGs and normalize the read count. The Pearson correlation of each sample showed that all samples were highly correlated.

The R package clusterProfiler^6^ was used for Go function and GSEA KEGG analysis. Additionally, Gene Set Variation Analysis (GSVA)^7^ was employed for GO function and KEGG pathway analysis using the R package GSVA, and the limma package (version 3.25.15; [bioinf.wehi.edu.au/limma](http://bioinf.wehi.edu.au/limma))^8^ was used to detect the differentially enriched functions and pathways (adjusted p-value < 0.05).

Raw data have been deposited in the National Center for Biotechnology Information Gene Expression Omnibus DataSets under accession number GSE150368.

*Bioinformatics methods used to evaluate the immune infiltration level of PCa*

The R package estimate was used to evaluate the stromal score, immune score and tumor purity of PCa by using the Estimation of STromal and Immune cells in MAlignant Tumor tissues using Expression data (ESTIMATE) method^9^. Single-sample gene set enrichment analysis (ssGSEA) (R package “GSVA”)^10^ was used to computationally infer the infiltration level of specific immune cell types using RNA-seq data. We used 22 immune-related cell types from the literature that included three immunity classifications: pro-tumor suppression immunity, antitumor immunity and other components^11^.

*Weighted correlation network analysis*

WGCNA is an algorithm used in gene coexpression network identification by high-throughput expression profile mRNAs with different traits^12^. First, by calculating the correlations of top 8,269 variation genes, a matrix of similarity was constructed . Second, by using the pickSoftThreshold function in the R WGCNA package, an appropriate soft-thresholding power β was selected. Then this soft-thresholding power was used to increase the co-expression similarity and achieve scale-free topology. Third, the adjacency was transformed into a topological overlap matrix (TOM) using TOM similarity. Then, the corresponding dissimilarity (dissTOM) was also calculated. Fourth, by using the dynamic tree cutting methods, co-expression gene modules were identified with the following major parameters: 1) maxBlockSize of 20000. 2) minModuleSize of 30 and 3) deepSplit of 2. The module eigengene (ME), which was the first principal component (PC) of each module’s gene expression matrix, was obtained by WGCNA to represent the expression profiles of module genes^13^. Highly similar modules with the height of ME in the clustering lower than 0.25 were merged together. A clustering dendrogram was used to display the results of dynamic tree cutting and merging.

We followed the steps of data processing as outlined in the Horvath Lab UCLA protocol (<https://horvath.genetics.ucla.edu/> html/CoexpressionNetwork/Rpackages/WGCNA/Tutorials/).

*Immunohistochemistry and evaluation of immunostaining*

Antibodies against CD8 (Proteintech Group, Inc. Wuhan, China; Dilution: 1:800), SOCS3 (Proteintech Group, Inc. Wuhan, China; Dilution: 1:100), ZFP36 (Proteintech Group, Inc. Wuhan, China; Dilution: 1:100) and JUNB (Proteintech Group, Inc. Wuhan, China; Dilution: 1:100) were used to identify the corresponding protein expression. Tissue microarrays were constructed with 1.0-mm tissue cores. Briefly, before incubation with the corresponding primary antibodies and biotinylated secondary antibodies (Vector, Burlingame, CA), these sections were deparaffinized, hydrated, and antigen retrieved.

Slides were evaluated by observers who were blinded to their origin on an inverted Olympus microscope with the assistance of –Image-Pro Plus 6.0 image processing software (Media

Cybernetics Inc). When scoring the immunoreactivity of SOCS3, ZFP36 and JUNB, we used the H-score, a semicontinuous variable scoring system that has been used in many previous studies^14,15^. In detail, the percentage of immunostaining and the staining intensity were recorded. The percentage of immunostaining was measured using Image-Pro Plus 6.0 software (Media Cybernetics Inc.). The percentage of positive cells in each core was scored between 0% and 100%. For staining intensity, strong, moderate and weak intensity was defined as dark brown, tan and light yellow staining areas, and no staining was defined as the same staining as the negative control. When grading the staining intensity, we used the optical density value generated by Image-Pro Plus as an important reference. The H-score was calculated using the following formula: (percentage of cells of weak intensity×1) + (percentage of cells of moderate intensity×2) + (percentage of cells of strong intensity×3). As a consequence, the H-score provided a semicontinuous score between 0 and 300 for each core. CD8+ individual infiltrating cells were quantified as the numbers of cells per unit area of the entire sections and changed into density as cells/mm^2^.

Immunoreactivity was independently assessed by two investigators who were blinded to the clinicopathological data. The mean value of the scores assessed was taken as the final result. If their scores differed widely, the values were discussed until an agreement was reached.

Reference

1. Kim D, Langmead B, Salzberg SL. HISAT: a fast spliced aligner with low memory requirements. Nat Methods 2015; 12(4):357-60 doi:10.1038/nmeth.3317
2. Haas BJ. STAR-Fusion code and documentation on GitHub 2019. Available from: https://github.com/STAR-Fusion/STAR-Fusion/wiki.
3. Anders S, Pyl PT, Huber W. HTSeq–a python framework to work with high-throughput sequencing data. Bioinformatics 2015;31(2):166–9 doi:10.1093/bioinformatics/btu638
4. Pertea M, et al. StringTie enables improved reconstruction of a transcriptome from RNA-seq reads. Nat. Biotechnol*.* 2015;33(3):290–5 doi: 10.1038/nbt.3122
5. Love MI, Huber W, Anders S. Moderated estimation of fold change and dispersion for RNA-seq data with DESeq2. Genome Biol 2014; 15(12):550 doi:10.1186/s13059-014-0550-8
6. Yu G, Wang LG, Han Y, He QY. clusterProfiler: an R package for comparing biological themes among gene clusters. OMICS 2012;16(5):284–287. doi: 10.1089/omi.2011.0118.
7. Hänzelmann S, Castelo R, Guinney J. GSVA: gene set variation analysis for microarray and RNAseq data. BMC Bioinformatics 2013;14:7 doi:10.1186/1471-2105-14-7
8. Ritchie ME, et al. limma powers differential expression analyses for RNA-sequencing and microarray studies. Nucleic Acids Res. 2015;43(7):e47 doi:10.1093/nar/gkv007
9. Yoshihara K, et al. Inferring tumour purity and stromal and immune cell admixture from expression data. Nat Commun 2013;4:2612 doi: 10.1038/ncomms3612.
10. Angelova M, et al. Characterization of the immunophenotypes and antigenomes of colorectal cancers reveals distinct tumor escape mechanisms and novel targets for immunotherapy. Genome Biol. 2015;16:64 doi:10.1186/s13059-015-0620-6
11. Bindea G, et al. Spatiotemporal dynamics of intratumoural immune cells reveal the immune landscape in human cancer. Immunity 2013;39(4):782–795 doi: 10.1016/j.immuni.2013.10.003
12. Langfelder P, Horvath S. WGCNA: an R package for weighted correlation network analysis. BMC Bioinformatics 2008;9:559 doi: 10.1186/1471-2105-9-559.
13. Langfelder P, Horvath S. Eigengene networks for studying the relationships between co-expression modules. BMC Syst Biol. 2007;1:54. doi: 10.1186/1752-0509-1-54
14. Akfirat C, et al. Tumour cell survival mechanisms in lethal metastatic prostate cancer differ between bone and soft tissue metastases. J Pathol 2013; 230(3):291-7 doi: 10.1002/path.4180
15. Lotan TL, et al. PTEN protein loss by immunostaining: analytic validation and prognostic indicator for a high risk surgical cohort of prostate cancer patients. Clin Cancer Res 2011;17(20):6563-73 doi: 10.1158/1078-0432
