# Supplementary material for: Immune signature driven by ADT-induced immune microenvironment remodeling in prostate cancer is correlated with recurrence-free survival and immune infiltration
Source: Cell Death Dis. 2020 Sep 19;11(9):779. doi: 10.1038/s41419-020-02973-1 (PMC7502080; doi:10.1038/s41419-020-02973-1)
Supplement: Supplementary file 2 — Supplementary Figure Legends [file 41419_2020_2973_MOESM2_ESM.docx]

## Supplementary Figure Legends

**Supplementary Figure 1: A**: PCA score plot of all samples group by their phenotypes. PCA score plot of 1) PCa samples before and after ADT; 2) Precancerous samples before and after ADT; 3) PCa and precancerous samples pre ADT; 4) PCa and precancerous samples post ADT. **B:** Representative GSEA KEGG pathways in PCa tissues response to ADT. **C:** Representative GO functions of DEGs in paracancerous benign samples response to ADT. GO categories are grouped according to functional theme**. D:** Representative GSEA KEGG pathways in paracancerous benign tissues response to ADT.

**Supplementary Figure 2: A and B:** Representative enriched GO functions (A) and KEGG pathways (A) of genes in bisque4 module. **C:** Pearson correlation coefficient of 5 hub genes in TCGA cohort. **D:** Pearson correlation coefficient between 5 hub genes and the infiltration level of 22 immune cell types in the PCa TIM in TCGA cohort.

**Supplementary Figure 3:** Immune signature score based subtypes was Association with the immune infiltration in ICGC cohort. **A:** Left: Heatmap of 22 related cell types across 144 PCa samples distinguished three immunological patterns in ICGC cohort. Right: The box plot shows immune signature score between the three subtypes in ICGC cohort. **B:** Immune score calculated by ESTIMATE in immune high and low subtypes. **C:** Difference of infiltration level of 22 immune cell types between immune high and low subtypes and the correlation of infiltration level of 22 immune cell types and immune signature score. **D:** Expression of immune related genes and immune checkpoints genes in immune high and low subtypes. **E:** Representative enriched GO functions of DEGs between immune high and low subtype. GO categories are grouped according to functional theme**. F:** Representative enriched GSEA KEGG pathways enriched in immune high subtype.
