# Supplementary material for: Immune signature driven by ADT-induced immune microenvironment remodeling in prostate cancer is correlated with recurrence-free survival and immune infiltration
Source: Cell Death Dis. 2020 Sep 19;11(9):779. doi: 10.1038/s41419-020-02973-1 (PMC7502080; doi:10.1038/s41419-020-02973-1)
Supplement: Supplementary file 3 — Supplementary Table Legends [file 41419_2020_2973_MOESM3_ESM.docx]

**Supplementary Table Legends**

Supplementary Table S1: Demographic information of patients

Supplementary Table S2: Differentially expressed genes of PCa and paracancerous tissues response to ADT (Post ADT vs Pre ADT)

Supplementary Table S3: List of Fusion genes

Supplement Table S4: The enriched Go functions and KEGG pathways of the Differentially expressed genes in PCa and paracancerous tissues response to ADT.

Supplementary Table S5: Summary of multivariate cox analysis for prediciting PSA recurrence
